# A new species of the lichenised genus *Anamylopsora* (Baeomycetaceae, Baeomycetales) from Tengger Desert of China

**DOI:** 10.3897/mycokeys.110.28168

**Published:** 2018-11-05

**Authors:** Ya-Bo Zuo, Da-Le Liu, Cui-Xin Li, Yu-Hui Chen, Xin-Li Wei

**Affiliations:** 1 College of Life Sciences, Southwest Forestry University, Kunming, Yunnan 650224, China Institute of Microbiology, Chinese Academy of Sciences Beijing China; 2 State Key Laboratory of Mycology, Institute of Microbiology, Chinese Academy of Sciences, Beijing 100101, China Southwest Forestry University Kunming China; 3 University of Chinese Academy of Sciences, Beijing 100049, China University of Chinese Academy of Sciences Beijing China

**Keywords:** Lichen, morphology, phylogeny, taxonomy, Tengger Desert

## Abstract

The monotypic lichenised genus *Anamylopsora* (Baeomycetaceae, Baeomycetales), with its single species *A.pulcherrima*, is distributed in the arid areas of the Northern Hemisphere, including China. In this paper, we introduce another species new to science, *Anamylopsorapruinosa*. The new species is characterised by a densely pruinose upper surface, abundantly thick and strong rhizines and terricolous habitat. It is also strongly supported by the phylogenetic and species delimitation analyses based on nrDNA ITS sequences, in which *A.pruinosa* forms well-supported clade separated from *A.pulcherrima*.

## Introduction

The monotypic genus *Anamylopsora* Timdal was established in 1991 (Timdal 1991), based on the species *Anamylopsorapulcherrima* (Vain.) Timdal. The species was previously excluded from *Psora* Hoffm., as *Psorapulcherrima* (Vain.) Elenkin, due to having, for example, a non-amyloid tholus and hymenial gelatine and is temporarily placed in the collective genus *Lecidea* Ach. (Timdal 1984). Together with *Lecidea*, the genus *Anamylopsora* was included in the family Lecideaceae Chevall., although it was observed to be more similar to Trapeliaceae M. Choisy ex Hertel in the ascus structure (Timdal 1991). Lumbsch et al. (1995) established a monotypic family Anamylopsoraceae in the Agyriineae (Lecanorales) based on the ascus structure, chemistry, pycnidial structure and ascoma ontogeny, comparing with all the morphologically similar or related families, such as Agyriaceae Corda, Baeomycetaceae Dumort., Icmadophilaceae Triebel, Lecideaceae and Psoraceae Zahlbr.

Later, the family Anamylopsoraceae was synonymised with the Baeomycetaceae based on multigene phylogenetic analysis and the genus *Anamylopsora* is currently included under Baeomycetaceae (Baeomycetales) (Resl et al. 2015), together with *Ainoa* Lumbsch & I. Schmitt, *Baeomyces* Pers. and *Phyllobaeis* Kalb & Gierl (Jaklitsch et al. 2016). The family is distant from *Psora* (Lecanorales) and *Lecidea* (Lecideales) (Resl et al. 2015). Hence, *Anamylopsorapulcherrima* belongs to a monotypic genus, but not monotypic family.

*Anamylopsorapulcherrima* is saxicolous, common in the arid areas of the Northern Hemisphere, including Asia (China, Iran, Kirgizstan, Mongolia, Nepal, Japan), Russia and U.S.A. (Davydov 2014; Inoue 2010; Moniri and Sipman 2009; Timdal 1991; Zhurbenko 2010). During our field survey in the arid region of the Northwest China, a new species of *Anamylopsora* was found in Tengger Desert with the characters of terricolous habitat, dense pruina and abundant rhizines. The purpose of this study is to describe the new member of the previously monotypic genus. Phylogenetic and species delimitation analyses based on nrDNA ITS sequences are also provided.

## Materials and methods

### Phenotypic analysis

All the six specimens of the new species of *Anamylopsora* were collected from one locality in the Ningxia Hui Autonomous Region of China, close to the Inner Mongolia Autonomous Region and are preserved in the Lichen Section of Herbarium Mycologicum Academiae Sinicae (HMAS-L). A dissecting microscope (Zeiss Stemi SV11) and compound microscope (Zeiss Axioskop 2+) were used for the study of morphology and anatomy. Standardized thin-layer chromatography (TLC, solvent system C) was used for the identification of secondary metabolites (Culberson 1972; Culberson and Kristinsson 1970; Orange et al. 2001).

### DNA extraction, amplification and sequencing

DNA was extracted from six fresh specimens of *Anamylopsora* (Table 1) following the modified CTAB method (Rogers and Bendich 1988). The internal transcribed spacer of nuclear ribosome DNA (nrDNA ITS) was chosen as the genetic marker. Primers LR1 (Vilgalys and Hester 1990) and ITS1 (White et al. 1990) were used. Reactions were carried out in 50 µl reaction volume and the components used were 3 µl total DNA, 1 µl each primer (10 µM), 25 µl 2×Taq MasterMix and 20 µl ddH_2_O. PCR amplifications were carried out in a Biometra T-Gradient thermal cycler, following conditions: initial heating step for 5 min at 95 °C, followed by 35 cycles of 30 s at 94 °C, 30 s at 56 °C, and 1 min 30 s at 72 °C; a final extension step of 8 min at 72 °C was added, after which the samples were kept at 4 °C. Negative controls were prepared for each amplification series. PCR products were purified using a gel purification kit (Shanghai Huashun Bioengineering Corporation, China) following the manufacturer’s instructions.

**Table 1.** Specimens of *Anamylopsora* from China and taxa used in the phylogenetic analysis in this study.

Taxon	Voucher specimens	GenBank No.
* Anamylopsora pruinosa *	XL2017133 (HMAS-L-141383)	MH558055*
* A. pruinosa *	ZW2018064 (HMAS-L-141384)	MH558056*
* A. pruinosa *	ZW2018099 (HMAS-L-141386)	MH558057*
* A. pruinosa *	ZW2018100 (HMAS-L-141385)	MH558058*
* A. pruinosa *	ZW2018101 (HMAS-L-141388)	MH558059*
* A. pruinosa *	ZW2018102 (HMAS-L-141387)	MH558060*
* A. pulcherrima *	Russia, Yakutia, 1992, Zhurbenko (ESS)	AF274089
* A. pulcherrima *	Zhurbenko 023, 2002(GZU)	KR017064
* Ainoa mooreana *	Nordin 7455 (UPS)	KJ462262
* Ainoa mooreana *	Thor 28340 (UPS)	KJ462263
* Anzina carneonivea *	Austria, Tyrol, 1996, Guderley & Heibel (ESS)	AF274077
* Baeomyces placophyllus *	XZ12147 (SDNU)	KT601493
* B. rufus *	yn138 (SDNU)	KT601494
* Phyllobaeis imbricata *	852	HQ650635
* Psora crenata *	Rui & Timdal SA11/02 (O)	MG677191
* Tephromela armeniaca *	u267	AY541278
* Trapelia coarctata *	Orange 23617 (NMW)	KY797787

* = sequences newly generated for this study by the authors

### Sequence alignment and phylogenetic analysis

PCR products were sequenced using the ABI 3730 XL Sequencer by Shanghai BioSune Corporation of China. Except sequences of the new species, the sequences of another species in *Anamylopsora*, *A.pulcherrima* and eight species in seven genera related as outgroups, i.e. *Ainoamooreana*, *Anzinacarneonivea*, *Baeomycesplacophyllus*, *B.rufus*, *Phyllobaeisimbricata*, *Psoracrenata*, *Trapeliacoarctata* and *Tephromelaarmeniaca*, were downloaded from GenBank. The sequences were aligned using ClustalW Multiple Alignment (Thompson et al. 1994) in BioEdit 7.2.5 (Hall 1999). The programme Gblocks v0.91b (Castresana 2000; Talavera and Castresana 2007) was used to delimit and remove regions of alignment uncertainty, using options for a “less stringent” selection on the Gblocks web server (http://molevol.cmima.csic.es/castresana/Gblocks_server.html). The alignment was subjected to a maximum likelihood (RAxML) analysis and nodal support was assessed using 1000 bootstrapping pseudo-replicates with RAxML-HPC v. 8.2.6 (Stamatakis 2014) and MrBayes v.3.2.6 (Huelsenbeck and Ronquist 2001; Ronquist and Huelsenbeck 2003) on the Cipres Science Gateway (http://www.phylo.org). In the ML and Bayesian analyses, substitution models for ITS were estimated using jModelTest-2.1.9 (Darriba et al. 2012; Guindon and Gascuel 2003). Based on these results, we used the TrN + I + G model with 1000 pseudoreplicates in the ML analysis and the TrN + G model in the Bayesian analysis. Two parallel Markov chain Monte Carlo (MCMC) runs were performed in MrBayes, each using 8 million generations and sampling every 1000 steps. A 50% majority-rule consensus tree was generated from the combined sampled trees of both runs after discarding the first 25% as burn-in. Tree files were visualised with FigTree v.1.4.2 (http://tree.bio.ed.ac.uk/software/figtree/). The intraspecific and interspecific genetic distances of the *Anamylopsora* species were also calculated and compared.

### Species delimitation analyses

Two species delimitation methods were used to circumscribe species boundaries within the genus *Anamylopsora* – “Automatic Barcode Gap Discovery” (ABGD) (Puillandre et al. 2012) and a Bayesian implementation of the Poisson tree process model (bPTP) (Zhang et al. 2013). For ABGD we used default parameters except for using a Pmax at 0.01 and a relative gap width of 1.5, with the model Jukes-Cantor (JC69). The bPTP model is intended for delimiting species in these single-locus molecular phylogenies, and provides an objective approach for delimiting putative species boundaries that are consistent with the phylogenetic species criteria. We used the bPTP web server (http://species.h-its.org, Zhang et al. 2013) to delimit putative species groups using the ITS topology as the input tree and implementing default settings.

## Results

### Phylogenetic analysis

**Figure 1.**
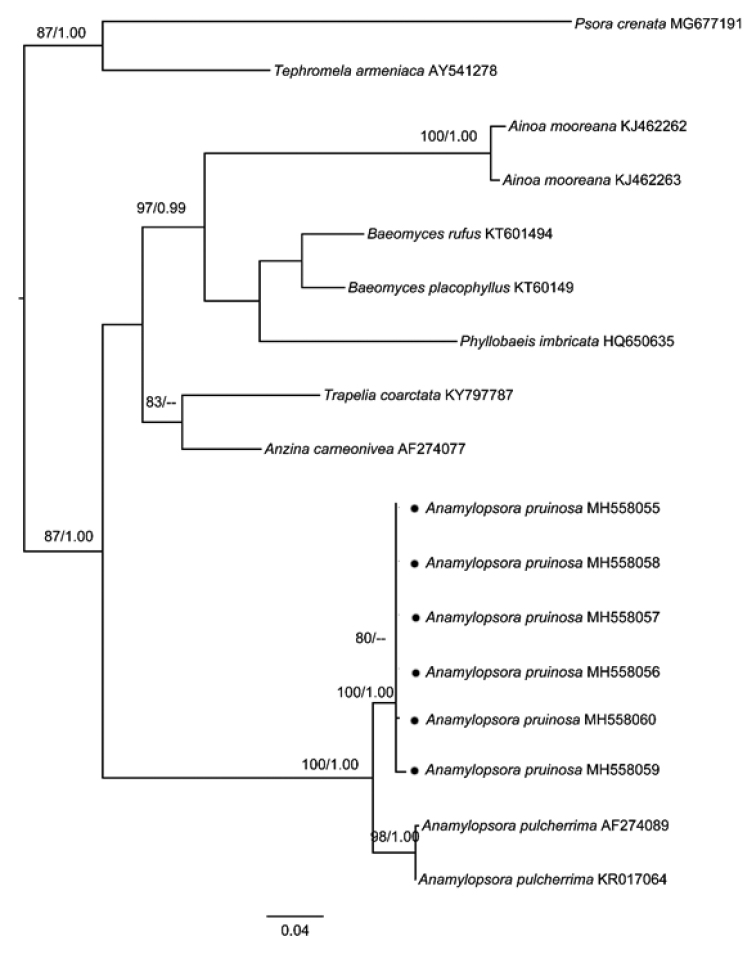
The maximum likelihood tree of *Anamylopsora* species based on the ITS sequences. The numbers in each node represent bootstrap support (BS) and posterior probability (PP) values. Bootstrap values ≥ 75 and posterior probability values ≥ 0.95 were plotted on the branches of the RAxML tree. Except for the new species *Anamylopsorapruinosa*, marked by the solid circle ‘●’, all the other sequences were downloaded from GenBank. Scale bar: 0.04 substitution per site.

The aligned matrix contained 431 unambiguous nucleotide position characters for ITS. The phylogenetic tree included 10 taxa representing five families from ca. four different orders and is illustrated in Fig. 1. *Anamylopsora* formed a well-supported (BS=100, PP=1.00) monophyletic clade, within which the new species obviously separated from *A.pulcherrima*. The genetic distances (Table 2), based on nrDNA ITS sequences within *Anamylopsora*, showed that the intraspecific distance range was 0.00–0.01, while the interspecific distance range was 0.04–0.05, also indicating they are two different species.

**Table 2.** Intraspecific and interspecific genetic distances range of the species in this study.

Taxon	1	2	3	4	5	6	7	8	9	10	11	12	13	14	15	16
1 *Anzinacarneonivea*AF274077																
2 *Baeomycesplacophyllus*KT601493	0.15															
3 *B.rufus*KT601494	0.14	0.06														
4 *Trapeliacoarctata*KY797787	0.10	0.16	0.15													
5 *Diploschistesdiacapsis*KX545503	0.30	0.32	0.33	0.31												
6 *D.muscorum*KX545481	0.29	0.33	0.32	0.30	0.02											
7 *Tephromelaarmeniaca*AY541278	0.18	0.20	0.20	0.21	0.36	0.35										
8 *Psoracrenata*MG677191	0.26	0.27	0.30	0.32	0.46	0.46	0.23									
9 *Romjularialurida*KF683091	0.16	0.19	0.20	0.19	0.37	0.37	0.21	0.27								
10 *Anamylopsorapulcherrima*KR017064	0.17	0.16	0.19	0.22	0.30	0.30	0.23	0.32	0.24							
11 *A.pulcherrima*AF274089	0.17	0.16	0.19	0.22	0.30	0.30	0.23	0.32	0.24	0.00						
12 *A.pruinosa*MH558055	0.18	0.17	0.19	0.23	0.31	0.31	0.21	0.32	0.25	0.04	0.04					
13 *A.pruinosa*MH558056	0.18	0.17	0.19	0.23	0.31	0.31	0.21	0.32	0.25	0.04	0.04	0.00				
14 *A.pruinosa*MH558057	0.18	0.17	0.19	0.23	0.31	0.31	0.21	0.31	0.25	0.04	0.04	0.00	0.00			
15 *A.pruinosa*MH558058	0.18	0.17	0.19	0.23	0.31	0.31	0.21	0.31	0.25	0.04	0.04	0.00	0.00	0.00		
16 *A.pruinosa*MH558059	0.19	0.18	0.20	0.24	0.33	0.33	0.22	0.32	0.25	0.05	0.05	0.01	0.01	0.01	0.01	
17 *A.pruinosa*MH558060	0.19	0.18	0.20	0.23	0.32	0.32	0.21	0.32	0.26	0.04	0.04	0.00	0.00	0.00	0.00	0.01

### Species delimitation analyses

The ABGD analysis based on nrDNA ITS, provided evidence supporting *A.pruinosa* and *A.pulcherrima* as two different species (P = 0.001-0.01). The tree-based bPTP analysis also suggested two species (tree not shown) and within *A.pruinosa* group, the individuals coll. nos ZW2018102 and ZW2018101 clustered outermost, separating from other four samples, i.e. coll. nos. XL2017133, ZW2018064, ZW2018099 and ZW2018100.

### Taxonomy

#### Anamylopsora pruinosa

Taxon classificationFungiBaeomycetalesAnamylopsoraceae

D.L. Liu & X.L. Wei sp. nov.

Figures 2a–i

##### Diagnosis.

The species is characterised by densely pruinose upper surface, abundantly thick and strong rhizines and terricolous habitat.

##### Type material.

CHINA. Ningxia: Zhongwei City, Ciu Liu Gou. 37°24'34.92"N, 104°35'8.66"E, 1577 m alt., on sandy soil, 15 July 2017, D.L. Liu & R. D. Liu XL2017133 (HMAS–L–141383– holotype).

##### Description.

Thallus squamulose, 2–6 cm diam., terricolous, tightly adnate to the substrate. Squamules 2–3 mm diam., more or less imbricate, with areolate crust-like centre and slightly ascending and crenate margin. Upper surface densely pruinose, occasionally naked part khaki, dull to slightly shiny. Lower surface pale brown near the margin, mostly absence of well-developed cortex. Rhizines abundant, ecorticate, simple to branched, 4–6.5 mm long, 0.5–0.8 mm thick. Outer layer of upper cortex pale brown, ca. 50 µm high; inner layer of cortex colourless, 125–150 µm high. Photobiont layer continuous, 50–150 µm high; algal cells green, unicellular. Medulla 112.5–250 µm high, containing pale brown crystals. Lower cortex brownish, 15–17.5 µm high. Apothecia lecideine, marginal, 0.5–2 mm diam., dark brown to black, occasionally cracked, dull, epruinose. Epithecium dark brown, ca. 12.5 µm high. Hymenium colourless, 75–100 µm high, hemi-amyloid; asci clavate, 50–125 × 7.5–12.5 µm, surrounded by an amyloid sheet; tholus more or less well developed, non-amyloid. 4–8 ascospores per asci, i.e. 4, 5, 6, 8; ascospores simple, subglobose, colourless, 7.5–10 µm diam.; paraphyses weakly conglutinated, simple, with slightly thickened and brown pigmented apical cells. Pycnidia marginal, subglobose, dark brown to black, 275–425 × 275–375 µm; conidia shortly bacilliform, colourless, 3.75–5 × 1.25–2.5 µm.

##### Chemistry.

Alectorialic and barbatolic acids.

##### Habitat and distribution.

On the surface of sand soil in the arid region of Northwest China, Tengger Desert, where the annual precipitation is under 200 mm.

##### Etymology.

Name refers the whole upper surface being densely pruinose.

##### Additional material examined.

CHINA. Ningxia: Zhongwei City, Ciu Liu Gou. 37°24'34.92"N, 104°35'8.66"E, 1577 m alt., on sandy soil, 1 June 2018, D.L. Liu et al. ZW2018064 (HMAS–L–141384), ZW2018099 (HMAS–L–141386), ZW2018100 (HMAS–L–141385), ZW2018101 (HMAS–L–141388), ZW2018102 (HMAS–L–141387).

##### Notes.

As known, *Anamylopsorapulcherrima* is saxicolous, growing on calciferous and non-calciferous rocks; upper surface epruinose or more rarely pruinose with more or less white pruinose margin (Timdal 1991). While the new species, *A.pruinosa*, is terricolous, growing directly on the surface of sandy soil, with thick and strong rhizines penetrating into the sand. On the other hand, the upper surface of *A.pruinosa* is densely white pruinose, occasionally very little part naked. Phylogenetic and species delimitation analyses based on ITS sequences (Fig. 1) also well supported that they are two different species.

**Figure 2.**
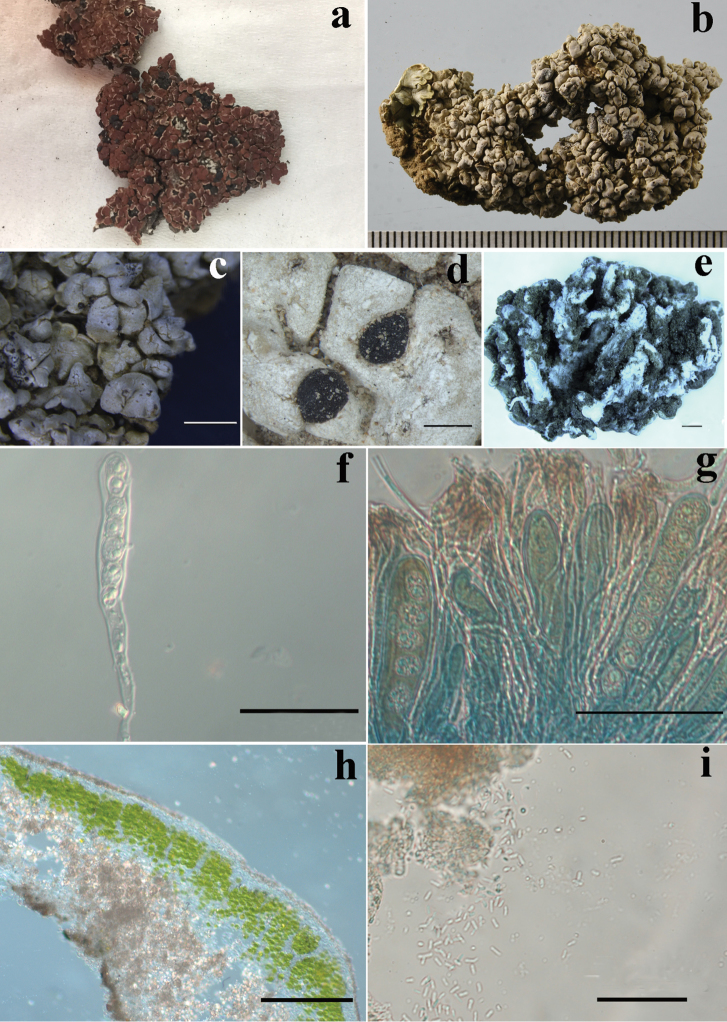
The new species *Anamylopsorapruinosa* (holotype, HMAS–L–141383). **a** Lichen thallus habit of *Anamylopsorapulcherrima* (C0090112F, F) **b** Lichen thallus habit of *Anamylopsorapruinosa* (holotype, HMAS–L–141383), scale in mm **c** Pruinose upper surface of the new species **d** The marginal apothecia of the new species **e** The abundant and thick and strong rhizines (white) at the lower surface **f** The asci with ascospores of the new species **g** The asci in iodine, showing the amyloid sheet **h** The thallus anatomical structure of the new species **i** The shortly bacilliform conidia of the new species. Scale bars: 0.2 mm (**c**); 0.5 mm (**d**); 0.95 mm (**e**); 50 µm (**f, g**); 200 µm (**h**); 50 µm (**i**).

## Discussion

Except for the diagnostic characters of the new species *Anamylopsorapruinosa*, most characters are accordant with the genus *Anamylopsora*, such as the habit of thallus (squamulose), type and location of apothecia (lecideine, marginal), weakly amyloid hymenium, asci with amyloid sheet, non-amyloid tholus, ascospores and conidia, and chemistry, etc. (Lumbsch et al. 1995; Timdal 1991). In addition, the phylogenetic analysis showed *Anamylopsora*, including the two species, to be monophyletic. The species delimitation analyses, including ABGD and bPTP, also supported *A.pruinosa* and *A.pulcherrima* as two separate species. Therefore, both the phenotypic observations and ITS sequences well supported the new species.

As the genus *Anamylopsora* was known to be monotypic before this study and only the species *A.pulcherrima* is accepted, there are, however, three synonyms, i.e. *Lecideapulcherrima* (Basionym), *Lecideahedinii* and *Lecideaundulata* (Timdal 1991). Based on the original description of *L.hedinii* and *L.undulata* (Magnusson 1940; 1944), the morphological characters of *L.undulata* was suspected to be most similar to the new species *A.pruinosa* in greyish-white lobes, densely pruinose and terricolous habitat, but *L.undulata* has much smaller conidia (2.5–3.5 × 0.8 µm), and ‘very large, reddish-brown apothecia’ (Magnusson 1940), which is much different from the new species, *A.pruinosa*, with larger conidia (3.75–5 × 1.25–2.5 µm) and not large (0.5–2 mm diam.) and black apothecia. Especially, we could not find any fresh materials of *L.undulata* and the corresponding DNA sequences were unavailable. Therefore, we could not judge whether the new species *A.pruinosa* is exactly the synonymized *L.undulata* with the knowledge we have. Fresh materials corresponding to *L.undulata*, are needed and then it may be possible to answer this question.

In the phylogenetic analysis, we included the other three genera, i.e. *Ainoa*, *Baeomyces* and *Phyllobaeis*, within Baeomycetaceae (Jaklitsch et al. 2016) and some related taxa previously mentioned, i.e. *Anzinacarneonivea* (Thelenellaceae, Incertae sedis order), *Psoracrenata* (Psoraceae, Lecanorales), *Trapeliacoarctata* (Agyriaceae, Baeomycetales) and *Tephromelaarmeniaca* (Lecanoraceae, Lecanorales) (Lumbsch et al. 2001a; b). The analyses well supported the monophyly of *Anamylopsora*. While obviously separating from the outgroup *Psoracrenata* and *Tephromelaarmeniaca* (Lecanorales), the relationship amongst other orders, i.e. Baeomycetales, Trapeliales and Incertae sedis (Thelenellaceae), were not clearly shown. More species and gene loci are needed to clarify the above-mentioned relationships.

In China, *Anamylopsorapulcherrima* has been found and reported in some arid regions, such as Inner Mongolia and Gansu (Magnusson 1940; 1944; Schneider 1979), and also in Tibet (Obermayer 2004), but all these known species grow on calciferous stone, meaning that it is saxicolous. We did not find the corresponding description about whether rhizines were present in this species and we also did not find obvious rhizines through observation of the specimen deposited in F (C0090112F). However, the terricolous new species, *A.pruinosa*, directly grows on the surface of sandy soil, tightly adnate to the substrate by the abundant, thick and strong rhizines, forming an important type of lichen crust in the desert area, possibly contributing to sand-fixation. Previously, we generally focused on the predominant genus *Endocarpon* (Verrucariaceae, Verrucariales) in the Tengger Desert (Yang and Wei 2008; Zhang et al. 2017) due to their sand-fixation ability by rhizines. Comparing to *Endocarpon* spp., *Anamylopsorapruinosa* may, however, have more and greater advantages in their type of rhizines. Therefore, it is necessary to pay more attention to some other advantageous species like *Anamylopsorapruinosa* and try to apply them in the sand control engineering (Wei 2005) in the near future.

## Supplementary Material

XML Treatment for Anamylopsora pruinosa
